# Effects of the Enriched Sports Activities-Program on Executive Functions in Italian Children

**DOI:** 10.3390/jfmk5020026

**Published:** 2020-04-23

**Authors:** Marianna Alesi, Giulia Giordano, Marcello Giaccone, Michele Basile, Sebastiano Costa, Antonino Bianco

**Affiliations:** 1 Department of Psychology, Educational Science and Human Movement, University of Palermo, 90128 Palermo, Italy; marianna.alesi@unipa.it (M.A.); antonino.bianco@unipa.it (A.B.); 2 Psychologist, Independent Researcher, 90036 Misilmeri (PA), Italy; 3 Study Center of University Sports Center Palermo (CUS Palermo), University of Palermo, 90128 Palermo, Italy; marcello.giaccone@unipa.it (M.G.); miter702@libero.it (M.B.); 4 Department of Psychology, University of Campania Luigi Vanvitelli, 81100 Caserta, Italy; sebastiano.costa@unicampania.it

**Keywords:** executive functions, physical activity, ESA-Program

## Abstract

Physical activity (PA) during childhood plays an important role in brain development. This role is played in both the structural domain, prefrontal cortex area, and in the functional domain, involving the higher cognitive functions, including the executive functions (EF). Working memory (WM), inhibition, and switching as fundamental EF were investigated in an Italian children sample before and after four months of an Enriched Sports Activities-Program (ESA-Program). EFs were assessed at pre-test and post-test using, respectively, the digit span test, the color word Stroop test, and the trail making test derived from Millisecond Software. The Italian sample was composed of 141 children aged 8.54 years. The intervention group (IG) was composed of 61 children and the control group (CG) of 80 children. Significant differences in WM (*p* < 0.05) were found in the IG following the ESA-Program intervention. Children’s performance improved both in forward digit span (FDS1 mean difference = 0.26; *p* = 0.04; FDS2 mean difference = 0.21; *p* = 0.01) and backward digit span (BDS2 mean difference = 0.14; *p* = 0.02). No significant improvements were observed for inhibition and switching processes (*p* > 0.05). Since this finding suggests that brain functioning is sensitive to lifestyle factors, such as PA, an essential goal for ESA-Program is to emphasize the importance of PA to enhance cognitive skills in childhood and prevent sedentary life.

## 1. Introduction

A physical and active lifestyle during childhood seems to be a powerful marker associated with brain development, in terms of brain structure and functioning, and, as a consequence, with cognitive development [1,2].

There is evidence supporting the close relationship between motor development and cognitive functions [3,4]. The crucial factor is assumed to be a causal link between regular physical activity (PA) and brain growth in the prefrontal cortical area [5]. This relationship finds a possible explanation in the executive functions (EFs) hypothesis according to which exercise training sessions influence a significant increase in gray matter volume [6,7], and greater white matter integrity [8]. This is related to a prolonged growth of myelination and connectivity between the age of 7 and early adulthood in prefrontal and frontal cortex areas [2,9].

Chaddock-Heyman et al. (2014) noted denser white matter among higher fit children compared to lower fit peers; this was associated with a significantly better functioning for memory, attention, and cognitive efficiency [10]. Esteban-Cornejo et al. (2017) examined the three main components of physical fitness: cardiorespiratory, motor, and muscular fitness [11]. Two of these, cardiorespiratory and speed-agility, were linked to greater gray matter volumes in cortical areas, specifically in frontal regions, and in subcortical areas in both normal weight and overweight/obese children [11]. In a more recent study [12], a positive association between the three components of physical fitness and white matter volume in overweight/obese children was also found. Physical fitness might improve integration between brain structures, which supported EFs [12]. Another recent study [13] found a relation between PA and white matter microstructure in overweight or obese children.

In particular, the enhancement of cortical network in frontal cortex area as a result of exercise training sessions improves children’s EFs, such as working memory (WM), speed and accuracy of processing, strategy employ, and inhibition process [2,9]. EFs were defined by Best as “the cognitive processes necessary for goal-directed cognition and behavior” [5] (p. 331). Three processes are considered as the most important: inhibition, switching, and WM [14]. Inhibition is the ability to control and repress an impulsive response; shifting is the ability to move from a stimulus or perspective to another one; WM is the ability to manipulate cognitive information held in mind [15].

Cross-sectional studies investigated the relationship between PA and EFs. They showed that most high-level cognitive processes, such as inhibition, switching, WM, and planning, were related to aerobic exercise in adulthood [16,17,18,19] and childhood [20,21,22,23]. Children with higher fitness levels showed better WM [24] and inhibition processes [20]; moreover, fitness in preadolescent children might be related to better executive control [21] and cognitive flexibility [22]. Motor skills, such as object control, have been related to visuospatial WM [23]. Previous studies focused on different EFs domains, and most of the findings confirmed the heterogeneity of the association between PA and different cognitive skills [18,19,20,21]. This heterogeneity of results clearly showed the necessity to examine different domains of EF to understand the interplay between motor and cognitive skills [18]. Nevertheless, intervention studies demonstrated that to take part in PA enriched by cognitive stimuli have better effects on EFs compared to PA without this enrichment [25,26,27,28]. The involvement in complex activities in terms of motor coordination may transfer executive function skills to other contexts [5].

Structured PA stimulates EFs such as focused attention, the memory of complex sequences, and the flexibility to adapt to unfamiliar tasks [29]. However, the maximum benefit to executive functioning can be achieved by those PA that, on the one hand, challenge executive functions, and on the other hand, also bring joy, sense of belonging and social inclusion, self-esteem, and motor and aerobic abilities, by enhancing, at the same time, physical fitness and skills [29,30,31]. Furthermore, not only the quantity of PA is important to improve EFs [29], but its qualitative nature [30].

Previous studies [20,21,22,23,24] clearly showed that exercise could be related to several domains of EF, and for this reason, this is an extremely relevant study of the role of training programs across the EF domains. This type of study could be very useful for understanding if there is a similar effect for all the domains of EF, or if some domains receive a stronger influence by exercise.

In line with these studies, the Enriched Sports Activities (ESA)-Program has been developed. It was an evidence-based practice exercise program cofounded by the Erasmus + Programme of the European Union (Key action: Sport-579661-EPP-1-2016-2-IT-SPO-SCP). The project was carried out in seven European Countries: Italy, Lithuania, Turkey, Germany, Portugal, Spain, and Croatia. It was developed over three years, from 2016 to 2019, and it involved a specialized practitioners team (coaches, sports scientists, and psychologists) and the establishment of a European network among families, practitioners, and schools. This study aims to examine the efficacy of the ESA-Program to enhance EFs over 4 months of intervention in an Italian children sample. We hypothesize that the intervention group children would have better EF performance than the control group.

## 2. Materials and Methods

### 2.1. Participants

The sample consisted of 141 Italian children with an average age of 8.54 years (SD = 1.23; age-range 7–14 years). Children were randomly assigned to two groups by a simple chance procedure. A randomly generated sequence of numbers from 1 to 2 was used to allocate each participant in the intervention group (IG) or the control group (CG). The IG was composed of 61 children (boys = 67.2%, girls = 32.8%; average age M = 8.34 years, SD = 1.57), and the CG was composed of 80 children (boys = 47.5%, girl = 52.5%, average age M = 8.69 years, SD = 0.87) (see Table 1). Initially, there were two groups of 85 participants, but immediately after the experimentation began, a few participants dropped out of the program for family reasons. At this stage, it was not possible anymore to re-balance the group members. The participants were recruited from two primary schools and from one sports center, the CUS (University Sports Centre) in Sicily, that provided their adhesion to the research project. The main inclusion criterion was the participants’ age, between 7 and 14 years, while the exclusion criterion was the presence of intellective disabilities or other kinds of disabilities. All the children’s parents took part in a preliminary meeting aimed at explaining the research aims, procedure, and details. Prior to the start of the study, each participant’s parents provided written informed consent and certification of suitability for non-competitive sports activities in the research. All were guaranteed the anonymity of their data. Teachers, coaches, and parents were introduced to the aims and objectives of the study.

### 2.2. Procedure

A schedule of pre-tests was agreed with the schools, and the teachers led their children to the testing site at the scheduled time. The administration took place in a quiet environment inside the schools or the sports center. Cognitive tests took about 40 min to be completed.

A longitudinal method was employed. It included a pre-test evaluation (T1) of EFs and post-test evaluation (T2) in which tests for EFs were repeated. After the pre-test phase, the structured ESA curriculum of 27 lessons was delivered over four months (January–April 2018), while the CG children received two times a week regular physical education activities, as scheduled by their school or sport curricula, for the same amount of time than IG. These activities consisted of exercises such as skipping (ahead, right, left), kick-running, fore-foot gait, rear-foot gait, sports balls, and dynamic balance.

The training duration of each module ranged from 15–25 min two times a week, where children were also given cognitive tasks during PA. Unfortunately, several children of the IG did not complete the ESA-Program activities, and they were not taken into account, explaining why IG had a smaller sample than the CG. Dropouts were 26 because they did not attend 80% of physical education lessons, or they had left the sport course, or they were absent when the post-test was carried out.

### 2.3. Measures

Before the cognitive assessment, a socio-demographic schedule was administered to collect children’s information about their age, education, school marks, and SES. The criteria for SES evaluation were parents’ number of children, parents’ qualification, and job. The medium–high status was derived by the parental qualification of high school or graduation and a job requiring a diploma or high qualification. The medium status was derived by parental qualification or middle school and a high-qualified job as a shop assistant or similar. The medium–low status was derived from the parental qualification of primary school and being qualified to be a housekeeper or student.

#### 2.3.1. Executive Functions Assessment

For the EF assessment, a battery of tests was used. This included the forward and backward digit span tests [32], the color word Stroop test [33], and trail making test [34] derived from the Millisecond Software. The digit span tests aim to measure WM. It is composed of two scales: the forward and the backward recall of numbers. Each scale, forward digit span (FDS) and backward digit span (BDS), is divided into two subscales: FDS maximum number of digits recalled correctly (FDS 1), FDS maximum number of digits recalled correctly before making two consecutive errors (FDS 2), BDS maximum number of digits recalled correctly (BDS 1), and BDS maximum number of digits recalled correctly before making two consecutive errors (BDS 2). The test includes 2 to 8 sequences of numbers with increasing level of difficulty to be repeated in the same order for FDS, and reverse order for BDS, until a correct response performance. Each digit was presented for one second on the laptop screen. The assessment was over after 14 trials. Scores were computed counting the number (n) of recalled digits in the presented order and the number of recalled digits in the reversed order.

The color word Stroop test assessed the ability to inhibit cognitive interference of word meaning on the naming of the color of the words. Words written in four colors (blue, black, red, and green) were shown. Children were asked to select the word color by keypress as fast as they could. There were three categories of stimuli: congruent trials (CTs) included words in which color word and the color print matched (i.e., RED painted in red color); incongruent trial (ITs) included words in which color word and color print were different (i.e., RED printed in blue color); Control Trials (ConTs) included only colored rectangles. Total trials were 84 derived by 4 colors (blue, black, red, green) × 3 color-stimulus congruency (congruent, incongruent, control) × 7 repetitions. Scores resulted in reaction time/accuracy differences to color-meaning congruent and color-meaning combinations. Reaction time was calculated in seconds (s).

The trail making test (TMT) measured visual attention and shifting skills. Children were asked to move the mouse in sequences to connect circles that represented stimuli belonging to different categories: numbers or letters or numbers and letters. Children were instructed to connect the circles as quickly as possible while still maintaining accuracy. The test was composed by four trials divided into two parts: Part A in which there were only numbers, the first trial with numbers from 1 to 5, the second trial with numbers from 1 to 25; and Part B in which there were numbers and letters, the third trial from 1 A to 5 E, the fourth trial from 1 A to 13 L. Children connected the circles in ascending pattern, but with the added task of alternating between the numbers and the letters. Number (n) of errors and time of execution, in seconds (s), were counted. Four scores were obtained: errors in numbers (TMT A Er), time in numbers (TMT A T), errors in numbers and letters (TMT B Er), and time in numbers and letters (TMT B T).

#### 2.3.2. The ESA-Program

The ESA-Program is an integrated (cognitive + motor) sports program composed of 27 units, with 2 sessions a week lasting about 15–25 min conducted by physical trainers. The implementation lasts 14 consecutive weeks. The ESA-Program aims to integrate typical sports activities, such as classical station sequences and kick-ups, with a combination of movements enriched by specific cognitive stimuli to strengthen basic executive functions, specifically, WM, inhibition, and shifting processes.

The motor program is subdivided into three domains of movement: pre-athletics, activities with balls, and dexterity/agility sequences. Pre-athletics section represents a series of smart circuits composed of exercises as skip-ahead; skip-right, skip-left kicked running. Activities with balls represent specific exercises used in sports balls as volleyball kick-ups both hands—upward/downward, and kick-ups both hands—downward/upward. Dexterity/agility sequences represent classical athletic drills such as balance on the line—ahead, balance on the line—behind, alternate leaps. Pre-athletics section represents a series of smart circuits composed of exercises as skip-ahead, skip-right, skip-left kicked running. Activities with balls represent specific exercises used in sports balls as volleyball kick-ups both hands—upward/downward, and kick-ups both hands—downward/upward. Dexterity/agility sequences represent classical athletic drills such as balance on the line—ahead, balance on the line—behind, alternate leaps. Each of the three domains of movement was composed of 3 units of beginner level, 3 units of intermediate level, and 3 units of advanced level with increasing level of motor difficulty and cognitive load. Each unit was composed of a baseline phase followed by an experimental phase. The experimental phase was enriched by the cognitive tasks derived by three cognitive domains: (1) inhibitory control, (2) WM, (3) shifting. The proportion of cognitive enrichment was 1:1, one cognitive task for each motor task.

The 27 units were scheduled per expertise level and per cognitive domain as follows:

- 9 units for each expertise level: 9 for beginner level, 9 for intermediate level, and 9 for advanced level.
- 3 units for each cognitive domains: 3 for inhibitory control, 3 for WM, and 3 for shifting.

Both phases, baseline and experimental, were performed by the IG children individually.

An example of ESA-Program unit for beginners (Unit 5) to increase WM was based on a course of 10 m lasting 15–25 min; the required movements were balance on the line ahead/behind, alternate leaps, quadruped position in a circuit. In the baseline phase (5′–7′), the trainer explained the circuit and the children had to reproduce the same movements. In the experimental phase (10′–15′), the exercises were the same as the baseline phase, but the athlete had to perform them in the opposite order, from the last to the first one. If the coach’s oral command was “balance on the line— ahead/behind”, the child’s expected movements were “balance on the line but behind/ahead” and so on (see Table 2).

### 2.4. Data Analysis

Data analyses were performed using SPSS Software for Windows, version 20.0 [35]. The level of significance was set with *p* < 0.05. As a preliminary step, to test the differences in variables at the pre-test between IG and CG, a series of univariate analyses of covariance (ANCOVA) was performed. The group was used as a fixed factor, and pre-test scores were defined as dependent variables. The covariate was children’s age because previous studies showed that EFs vary with age [36].

Then, to test the different variables at the post-test between IG and CG, a series of univariate ANCOVAs was performed. The group was used as a fixed factor, and post-test scores were defined as dependent variables; the covariates were children’s age and pre-test scores for each variable. ANCOVAs were performed for each cognitive test variable. ANCOVA is mathematical equivalent to an ANOVA for repeated measures model without group effect at pre-test and has more power with the randomized treatment assignment method [37,38].

Moreover, the effect size (Cohen’s *d*) was calculated for each variable. The effect size can be small (*d* = 0.20), medium (*d* = 0.50), and large (*d* = 0.80) [39] (p. 532). Examination of the Levene’s test was used to check for assumption before adopting ANCOVA, suggesting that the assumption has been sufficiently respected.

## 3. Results

### 3.1. Preliminary Analyses

Means (M) and standard deviations (SD) of WM, inhibition, and switching tests for IG and CG at pre and post-test are presented in Table 3.

Concerning the socio-economic status (SES), medium–high socioeconomic level was predominant (43.5%). As regards FDS 1, results showed significant differences for group, (*F* (1,138) = 5.78, *p* = 0.02, *η*^2^*_p_* = 0.04), and for the covariate age, (*F* (1,138) = 21.56, *p* < 0.001, *η*^2^*_p_* = 0.13). IG children reported higher scores than CG children.

For FDS 2, no significant differences were found for group, (*F* (1,138) = 3.43, *p* > 0.05, *η*^2^*_p_* = 0.02), the covariate age was significant (*F* (1,138) = 6.26, *p* = 0.01, *η*^2^*_p_* = 0.04). IG and CG children reported no significant differences in scores.

For BDS 1, significant differences were found for group, (*F* (1,138) = 4.67, *p* = 0.03, *η*^2^*_p_* = 0.03), and for the covariate age, (*F* (1,138) = 30.97, *p* < 0.001, *η*^2^*_p_* = 0.18). IG children reported higher scores than children of CG.

For BDS 2, no statistical significant differences were found for group, (*F* (1,138) = 0.96, *p* = 0.32, *η*^2^*_p_* = 0.007), while the covariate age was significant (*F* (1,138) = 29.48, *p* < 0.001, *η*^2^*_p_* = 0.18). No significant differences were found between IG and CG children’s scores.

As regards Stroop CT, no significant differences were found for group (*F* (1,138) = 0.49, *p* = 0.48, *η*^2^*_p_* = 0.004), the covariate age was significant (*F* (1,138) = 7.22, *p* = 0.01, *η*^2^*_p_* = 0.05). No significant differences were found between the two groups’ scores.

For Stroop IT, no significant differences for group were found (*F* (1,138) = 0.15, *p* = 0.69, *η*^2^*_p_* = 0.001), the covariate age was not significant (*F* (1,138) = 2.70, *p* = 0.10, *η*^2^*_p_* = 0.02). No significant differences between the two groups’ scores or changes linked to age.

For Stroop ConT, no statistical significant differences were found for group (*F* (1,138) = 0.08, *p* = 0.36, *η*^2^*_p_* = 0.006), while the covariate age was significant (*F* (1,138) = 14.07, *p* = 0.001, *η*^2^*_p_* = 0.09). This meant that there were no significant differences between IG and CG children’s scores.

As regards TMT A Err, no statistical significant differences for group were found (*F* (1,138) = 0.42, *p* = 0.52, *η*^2^*_p_* = 0.003), while the covariate age was significant (*F* (1,138) = 12.16, *p* = 0.001, *η*^2^*_p_* = 0.08). No significant differences were found between the two groups’ scores.

For TMT A T, no significant differences were found for group (*F* (1,138) = 2.02, *p* = 0.16, *η*^2^*_p_* = 0.01), while age as covariate was significant (*F* (1,138) = 21.53, *p* = 0.001, *η*^2^*_p_* = 0.14). No significant differences were found between IG and CG children’s scores.

For TMT B Err, no significant differences for group were found (*F* (1,138) = 0.02, *p* = 0.90, *η*^2^*_p_* = 0.001), while the covariate age was not significant (*F* (1,138) = 20.85, *p* = 0.001, *η*^2^*_p_* = 0.13). No significant differences between the two groups’ scores were found.

For TMT B T, no significant differences were found for group (*F* (1,138) = 0.59, *p* = 0.44, *η*^2^*_p_* = 0.004), while the covariate age was significant (*F* (1,138) = 37.47, *p* = 0.001, *η*^2^*_p_* = 0.21). No significant differences between the two groups’ scores were found.

Overall, results showed that prior differences between CG and IG existed for FDS1 (*p* = 0.02) and for BDS1 (*p* = 0.03) at pre-test (Table 3).

### 3.2. Post-Test Analyses

Means and standard deviations of EFs variables for IG and CG at the post-test are presented in Table 3.

With regard to FDS 1, ANCOVAs revealed significant differences associated to group, (*F* (1,137) = 4.33, *p* = 0.04, *η*^2^*_p_* = 0.03), to the covariate age, (*F* (1,137) = 4.67, *p* = 0.03, *η*^2^*_p_* = 0.03), and to pre-test (*F* (1,137) = 51.91, *p* < 0.001, *η*^2^*_p_* = 0.27). Age and pre-test were significant covariates. Children’s scores change at an increasing age, and they change from T1 to T2.

For FDS 2, significant differences were found for group, (*F* (1,137) = 6.21, *p* = 0.01, *η*^2^*_p_* = 0.04), for the covariates age, (*F* (1,137) = 14.40, *p* < 0.001, *η*^2^*_p_* = 0.09), and for pre-test (*F* (1,137) = 23.73, *p* < 0.001, *η*^2^*_p_* = 0.15). The covariates of age and pre-test resulted as statistically significant. It meant that children’s scores change at an increasing age, and they differed from T1.

For BDS 1, no significant differences were found for group, (*F* (1,137) = 0.54, *p* > 0.05, *η*^2^*_p_* = 0.004). The covariates age (*F* (1,137) = 4.04, *p* = 0.046, *η*^2^*_p_* = 0.03) and pre-test (*F* (1,137) = 60.54, *p* < 0.001, *η*^2^*_p_* = 0.31) were significant. No significant differences between IG and CG children’s scores were found.

For BDS 2, significant differences were found in associated to group (*F* (1,137) = 5.71, *p* = 0.02, *η*^2^*_p_* = 0.04), and to the covariates age (*F* (1,137) = 6.81, *p* = 0.01, *η*^2^*_p_* = 0.05) and pre-test (*F* (1,137) = 18.27, *p* < 0.001, *η*^2^*_p_* = 0.12). The two covariates of age and pre-test were significant. Children’s scores change at an increasing age, and they change from pre-test to post-test.

As regards Stroop CT, no significant differences were found for group (*F*(1,137) = 0.09, *p* = 0.77, *η*^2^*_p_* = 0.001), the covariate age was not significant (*F* (1,137) = 0.13, *p* = 0.72, *η*^2^*_p_* = 0.001), while the covariate pre-test was significant (*F* (1,137) = 26.76, *p* = 0.001, *η*^2^*_p_* = 0.16). It meant that there were no differences between the two groups’ scores, no differences between children of different ages, but the only difference found was between pre-test and post-test scores.

For Stroop IT, no significant differences were found for group (*F* (1,137) = 0.89, *p* = 0.35, *η*^2^*_p_* = 0.006), the covariate age was not significant (*F* (1,137) = 0.75, *p* = 0.39, *η*^2^*_p_* = 0.005) while the covariate pre-test was statistical significant (*F* (1,137) = 48.91, *p* = 0.001, *η*^2^*_p_* = 0.26). There were no differences between IG and CG children’s scores and no differences between younger and older children; the only difference was found between pre-test and post-test scores.

For Stroop ConT, no significant differences were found for group (*F* (1,137) = 0.07, *p* = 0.79, *η*^2^*_p_* = 0.001), the covariate age was not significant (*F* (1,137) = 0.14, *p* = 0.71, *η*^2^*_p_* = 0.001), while the covariate pre-test was significant (*F* (1,137) = 5.20, *p* = 0.02, *η*^2^*_p_* = 0.04). There were no differences between IG and CG children’s scores and no differences between younger and older children; the only difference was found between pre-test and post-test scores.

With regard to TMT A Err, no significant differences for group were found (*F* (1,136) = 0.67, *p* = 0.42, *η*^2^*_p_* = 0.005), the covariates age (F (1,136) = 3.04, p = 0.08, *η*^2^*_p_* = 0.02) and pre-test were not significant (*F* (1,136) = 2.79, *p* = 0.09, *η*^2^*_p_* = 0.02). No differences between IG and CG children’s scores were found, with no differences between younger and older children’s scores or between T1 and T2 scores.

For TMT A T, no significant differences were found for group (*F* (1,136) = 3.31, *p* = 0.07, *η*^2^*_p_* = 0.02), the covariate age was not significant (*F* (1,136) = 3.08, *p* = 0.08, *η*^2^*_p_* = 0.02), while the covariate pre-test was significant (*F* (1,136) = 65.92, *p* = 0.001, *η*^2^*_p_* = 0.33). It meant there were not found differences between IG and CG children’s scores, no differences between younger and older children’s scores. The only difference was found between pre-test and post-test scores.

For TMT B Err, no significant differences for group were found (*F* (1,136) = 1.19, *p* = 0.27, *η*^2^*_p_* = 0.01), the covariate age was not significant (*F* (1,136) = 0.08, *p* = 0.36, *η*^2^*_p_* = 0.01), while the covariate pre-test was significant (*F* (1,136) = 10.35, *p* = 0.002, *η*^2^*_p_* = 0.07). No differences were found between the two groups’ scores, no differences were found between younger and older children’s performance scores, but there were differences between pre-test and post-test scores.

For TMT B T, no significant differences were found for group (*F* (1,136) = 1.12, *p* = 0.29, *η*^2^*_p_* = 0.01), the covariate age was not significant (*F* (1,136) = 0.08, *p* = 0.37, *η*^2^*_p_* = 0.01), while the covariate pre-test was significant (*F* (1,136) = 4.43, *p* = 0.04, *η*^2^*_p_* = 0.03). No differences were found between the two groups’ scores, and no differences were found between younger and older children’s performance scores, but there were differences between pre-test and post-test scores.

## 4. Discussion

The study aimed to assess the effects of ESA-Program on EFs in an Italian children sample (average age *M* = 8.54 years). After four months of the ESA-Program intervention, only WM showed an improvement. On the whole, IG children’s scores at post-test were higher than CG children’s scores. No significant differences were found for inhibition and switching variables (*p* > 0.05). This result could be explained by the fact that the sample of this study is defined by a group of young children and that during childhood, EF domains tend to be less differentiated. Previous studies have shown that the EFs structure changed from a general domain to a specific differentiation in which WM could become distinguishable from inhibition and switching processes [40].

The findings showed that ESA-Program intervention led to improvements in WM abilities for the IG, the only group that received the intervention. Specifically, after 4 months of Enriched Sport Activities-Program intervention, the number of digits recalled correctly before making two consecutive errors improved, both for FDS and BDS. This suggested that children improved their ability to manipulate verbal information while in temporary storage [41] and remembered more items before making two consecutive errors. However, no such effect was found for BDS 1. The differences in scores linked to participants’ age indicated that older children performed better because EFs, specifically WM, improves with increasing age. Young children’s lower scores might be explained by an immaturity of the working networks; in fact, in this age period, WM is still developing [36].

This result is in line with previous studies that have shown that PA could improve EFs; specifically, children in the IG (aged 8–12 years), who participated in a PA program lasting 22 weeks, with 30-min sessions twice a week, showed significant improvement on verbal WM skills tested using digit span tests [42]. Another study showed WM improvements following exercise enriched with cognitive tasks in adolescents with average age of 12.5 for only 8 weeks of intervention with 20-min sessions; however, in this study, the Stenberg task was used to test WM [43]. Furthermore, another study has shown that moderate-intensity exercise and its consequent physiological arousal increase could improve WM both during and after exercise, even though they used the Stenberg test to measure WM [44].

As underlined by previous research, PA might affect the development of WM abilities by acting multiple pathways at different levels, from brain cells to social interaction [45]. Continuing PA was linked to larger brain volume in the prefrontal cortex area, supporting EFs [7,46] and improvements in the connectivity of brain networks [47,48]. Structured PA during childhood might have long-term positive effects on cognitive development [45]. Children with a higher level of PA showed superior WM abilities; on the contrary, low PA levels at primary school ages were associated with poorer cognitive performance in terms of WM [49]. Children’s engagement in PA at 6 years of age predicted WM performance 8 years later, while a sedentary lifestyle during childhood might negatively influence cognitive maturation in adolescence [45]. Recent research has shown that WM could be improved by any change in WM abilities after the exercise session, independently from the exercise intensity. However, improvements in WM following a single session of aerobic exercise depends on baseline WM function [50].

In this study, the ESA-Program intervention improved only WM, and although it was an unexpected result, this is mostly in line with previous studies [42,51]. Previous studies, in fact, have shown that PA did not influence all the EF domains uniformly, but that different types of PA interventions could have different consequences. Furthermore, another explanation could be the fact that EFs tend to differentiate more with adolescence and adulthood, while in children, EFs tend to be more like a mono-factorial structure [52,53,54]. For this reason, it is possible that the improvement in WM is representative of the general improvement of EF in a development stage where dissociations of EF components were not emerged [52,53,54].

The study presents some limitations; the first is linked to the cognitive assessment addressed to WM. In the future, more sophisticated measures could be used to assess different WM components, such as visuospatial WM and not only verbal WM. Furthermore, in this study, it was not possible to evaluate the change in fitness and the intervention fidelity. Without this type of information, it could be difficult to be sure that changes observed were independent of fitness changes or other variables. Future studies should try to integrate these aspects to have a more complete evaluation.

The strengths of this study are, first of all, the integration of the cognitive domain and motor domain in the same sports program. Second, the different ages of the sample in this study allowed us to compare different developmental phases, and note that with the increasing age, WM also increase.

In conclusion, this study showed the efficacy of Enriched Sports Activities-Program intervention, based on sports activities enriched with cognitive tasks, to improve the WM in children. This, in line with other studies, demonstrates that the involvement in PA enriched by cognitive stimuli has better effects on EFs compared to PA without this enrichment [25,26,27,28]. For these reasons, the ESA-Program guideline could be used in future studies and projects about children’s lifestyles to highlight the importance of a PA during childhood and to prevent sedentary behaviors [55].

## Figures and Tables

**Table 1:** Demographic information for the total sample, the control group (CG), and the intervention group (IG).

Demographics	Total Sample	Control Group	Intervention Group
*N*	141	80	61
Age (year)—Mean (SD)	8.54 (1.23)	8.69 (.87)	8.34 (1.57)
Gender			
Boys	56%	47.5%	67.2%
Girls	44%	52.5%	32.8%
Socio-economic status (%)			
Medium low	20.1%	27.8%	10%
Medium	34.5%	45.6%	20%
Medium high	45.3%	26.6%	70%

**Table 2:** Enriched Sports Activities-Program (ESA-Program) Unit 5 for beginners description.

ESA 5 Beginner Level					
Setting		Course 10 m			
Duration		15–25 Minutes			
Domain of Movement		Circuit		Balance on the line ahead/behind–Alternate leaps–Quadruped position	
Executive Function		Working Memory			
Baseline Phase					
Balance on the line—ahead/behind; ESA walk/Alternate leaps; Balance on the line—behind/ahead; Alternate leaps/ESA walk; Balance on the line—ahead/behind; ESA walk/Alternate leaps; Balance on the line—behind/ahead; Alternate leaps/ESA walk; Balance on the line—ahead/behind; ESA walk/Alternate leaps; Balance on the line—behind/ahead; Alternate leaps/ESA walk					
Experimental Phase					
Oral Command			Expected Movement		
Balance on the line—ahead/behind; ESA walk/Alternate leaps; Balance on the line—behind/ahead; Balance on the line—ahead/behind; ESA walk/Alternate leaps; Balance on the line—behind/ahead; Alternate leaps/ESA walk; Balance on the line—ahead/behind; ESA walk/Alternate leaps; Balance on the line—behind/ahead; Alternate leaps/ESA walk			Balance on the line—behind/ahead; Alternate leaps/ESA walk; Balance on the line—ahead/behind; ESA walk/Alternate leaps; Balance on the line—behind/ahead; Alternate leaps/ESA walk; Balance on the line—ahead/behind; ESA walk/Alternate leaps; Balance on the line—behind/ahead; Alternate leaps/ESA walk; Balance on the line—ahead/behind; ESA walk/Alternate leaps.		

**Table 3:** Means, standard deviations, and effect size for digit span test, Stroop, and trail making test (TMT) scores before and after ESA-Program intervention for CG and IG.

	Pre-Test (T1)		Post-Test (T2)		Post-Test Effect Size CG vs. IG
	CG (*n* = 80)	IG (*n* = 61)	CG (*n* = 80)	IG (*n* = 61)	
Outcome Variables	M (SD)	M (SD)	M (SD)	M (SD)	*D*
FDS1 (n)	4.94 (1.00)	5.21 (0.95)	5.1 (0.83)	5.47 (1.07)	0.37*
FDS2 (n)	4.53 (0.86)	4.74 (0.81)	4.57 (0.88)	4.95 (1.07)	0.43 **
BDS1 (n)	4.16 (0.99)	4.38 (1.00)	4.11 (0.95)	4.29 (1.08)	0.13
BDS2 (n)	3.75 (1.06)	3.79 (1.02)	3.6 (0.93)	3.93 (1.21)	0.42 *
Stroop CT (s)	133.87 (34.50)	140.25 (36.64)	121.73 (22.82)	125.24 (31.85)	0.05
Stroop IT (s)	155.19 (45.92)	160.28 (50.79)	141.41 (35.80)	149.40 (40.23)	0.16
Stroop ConT (s)	130.05 (28.67)	137.54 (30.84)	133.63 (125.75)	134.83 (33.45)	0.04
TMT A Err (n)	3.60 (2.71)	3.52 (3.14)	2.39 (2.08)	2.88 (3.05)	0.14
TMT A T (s)	13,731.06 (5216.95)	13,103.31 (4564.69)	10,614.89 (4570.09)	11,635.82 (5077.09)	0.31
TMT B Err (n)	7.63 (5.53)	8.33 (6.26)	6.2 (3.88)	5.80 (5.16)	0.19
TMT B T (s)	20,565.70 (8169.99)	22,967.07 (11,569.65)	18,034.01 (16,829.12)	16,597.41 (6504.81)	0.18

*Note*. FDS1 (forward digit span maximum number of digits recalled correctly); FDS2 (forward digit span maximum number of digits recalled correctly before making two consecutive errors); BDS1 (backward digit span maximum number of digits recalled correctly); BDS2 (backward digit span maximum number of digits recalled correctly before making two consecutive errors); Stroop CT (Stroop congruent); Stroop IT (Stroop incongruent); Stroop ConT (Stroop control); TMT A Err (trial making test Part A errors); TMT A T (trial making test Part A time); TMT B Err (trial making test Part B errors); TMT B T (trial making test Part B time); M (mean), SD (standard deviation); d (effect size); n (number); seconds (s). Cohen’s *d* is defined by calculating the mean differences between CG and IG in post-test values adjusted for age and pre-test values, and then the result is divided by the pooled SD (** *p* < 0.01 between IG and CG; * *p* < 0.05 between IG and CG). Cohen’s *d* of pre-test vs. post-test for CG without adjustment for covariates were FDS1 = 0.17; FDS2 = 0.04; BDS2 = 0.15. Cohen’s *d* of pre-test vs. post-test for IG without adjustment for covariates were FDS1 = 0.26; FDS2 = 0.22; BDS2 = 0.13.

## Abbreviations

ESA	Enriched Sport Activities
EF	executive functions
IG	Intervention group
CG	Control group
WM	WM
PA	physical activity
SES	socio-economic status
CUS	University Sport Centre
T1	pre-test evaluation
T2	post-test evaluation
FDS	Forward Digit Span
BDS	Backward Digit Span
FDS 1	Forward Digit Span maximum number of digits recalled correctly
FDS 2	Forward Digit Span maximum of digits recalled correctly before making two consecutive errors
BDS 1	Backward Digit Span maximum number of digits recalled correctly
BDS 2	Backward Digit Span maximum of digits recalled correctly before making two consecutive errors
CT	Congruent trials
IT	Incongruent trials
ConT	Control trials
TMT	Trail Making Test
TMT Err	Trail Making Test Errors
TMT T	Trail Making Test Time
n	Number
s	Seconds
ANCOVA	Analysis of Covariance
